# Hering on Typhoid Fever

**Published:** 1897-06

**Authors:** 


### HOMŒOPATHIC PHYSICIAN,

A MONTHLY JOURNAL OF

HOMŒOPATHIC MATERIA MEDICA AND CLINICAL MEDICINE.

"If our school ever gives up the strict inductive method of Hahnemann, we are lost, and deserve only to be mentioned as a caricature in the history of medicine."—CONSTANTINE HERING.

Vol. XVII. JUNE, 1897. No. 6.

### EDITORIAL.

HERING ON TYPHOID FEVER.—This masterpiece of the immortal Hering, which has been reprinted in THE HOMEOPATHIC PHYSICIAN, is finished. The last six pages completing it were published in the May number. It can now be removed from the different numbers of the journal by turning to the back of the number, turning up the points of the wire stitches with a pocket-knife, and lifting the desired pages out.

It was intended that a second part should be added containing a complete Concordance Repertory to the symptoms given, and with the addition of all the later pathogenetic indications that have accumulated since Dr. Hering published his monograph.

This work was to have been done by Dr. Charles B. Gilbert, of Washington, D. C., long intimately associated with Dr. Hering, and therefore the one best fitted for such a valuable addition.

Unfortunately Dr. Gilbert's health has been so poor as to preclude the possibility of carrying out his design, and so we are reluctantly obliged to end the book without it. The editor has not, however, relinquished the hope that Dr. Gilbert may yet be able to accomplish his object.

